# A spitting image: molecular diagnostics applied to saliva enhance detection of *Streptococcus pneumoniae* and pneumococcal serotype carriage

**DOI:** 10.3389/fmicb.2023.1156695

**Published:** 2023-04-17

**Authors:** Willem R. Miellet, Janieke van Veldhuizen, David Litt, Rob Mariman, Alienke J. Wijmenga-Monsuur, Tessa Nieuwenhuijsen, Jennifer Christopher, Rebecca Thombre, Seyi Eletu, Thijs Bosch, Nynke Y. Rots, Marianne Alice van Houten, Elizabeth Miller, Norman K. Fry, Elisabeth A. M. Sanders, Krzysztof Trzciński

**Affiliations:** ^1^Department of Pediatric Immunology and Infectious Diseases, Wilhelmina Children’s Hospital, University Medical Center Utrecht, Utrecht, Netherlands; ^2^Centre for Infectious Disease Control, National Institute for Public Health and the Environment (RIVM), Bilthoven, Netherlands; ^3^Respiratory and Vaccine Preventable Bacteria Reference Unit (RVPBRU), UK Health Security Agency, London, United Kingdom; ^4^Spaarne Gasthuis, Haarlem, Netherlands; ^5^Department of Infectious Disease Epidemiology, Faculty of Epidemiology and Population Health, London School of Hygiene and Tropical Medicine, London, United Kingdom; ^6^Immunisation and Vaccine Preventable Diseases Division, UK Health Security Agency, London, United Kingdom

**Keywords:** *Streptococcus pneumoniae* (pneumococcus), pneumococcal serotypes, pneumococcal carriage, saliva, quantitative PCR (qPCR)

## Abstract

**Background:**

Despite strong historical records on the accuracy of saliva testing, oral fluids are considered poorly suited for pneumococcal carriage detection. We evaluated an approach for carriage surveillance and vaccine studies that increases the sensitivity and specificity of pneumococcus and pneumococcal serotype detection in saliva samples.

**Methods:**

Quantitative PCR (qPCR)-based methods were applied to detect pneumococcus and pneumococcal serotypes in 971 saliva samples collected from 653 toddlers and 318 adults. Results were compared with culture-based and qPCR-based detection in nasopharyngeal samples collected from children and in nasopharyngeal and oropharyngeal samples collected from adults. Optimal C_*q*_ cut-offs for positivity in qPCRs were determined via receiver operating characteristic curve analysis and accuracy of different approaches was assessed using a composite reference for pneumococcal and for serotype carriage based on isolation of live pneumococcus from the person or positivity of saliva samples determined with qPCR. To evaluate the inter-laboratory reproducibility of the method, 229 culture-enriched samples were tested independently in the second center.

**Results:**

In total, 51.5% of saliva samples from children and 31.8% of saliva samples from adults were positive for pneumococcus. Detection of pneumococcus by qPCR in culture-enriched saliva exhibited enhanced sensitivity and higher agreement with a composite reference compared to diagnostic culture of nasopharyngeal samples in children (Cohen’s κ: 0.69–0.79 vs. 0.61–0.73) and in adults (κ: 0.84–0.95 vs. 0.04–0.33) and culture of oropharyngeal samples in adults (κ: 0.84–0.95 vs. −0.12–0.19). Similarly, detection of serotypes with qPCR in culture-enriched saliva exhibited enhanced sensitivity and higher agreement with a composite reference compared to nasopharyngeal culture in children (κ: 0.73–0.82 vs. 0.61–0.73) and adults (κ: 0.90–0.96 vs. 0.00–0.30) and oropharyngeal culture in adults (κ: 0.90–0.96 vs. −0.13 to 0.30). However, results of qPCRs targeting serotype 4, 5, and 17F and serogroups 9, 12, and 35 were excluded due to assays’ lack of specificity. We observed excellent quantitative agreement for qPCR-based detection of pneumococcus between laboratories. After exclusion of serotype/serogroup-specific assays with insufficient specificity, moderate agreement (κ 0.68, 95% CI 0.58–0.77) was observed.

**Conclusion:**

Molecular testing of culture-enriched saliva samples improves the sensitivity of overall surveillance of pneumococcal carriage in children and adults, but limitations of qPCR-based approaches for pneumococcal serotypes carriage detection should be considered.

## Introduction

*Streptococcus pneumoniae* (pneumococcus) is an important cause of community-acquired pneumonia (CAP) and invasive bacterial disease but also a common colonizer of the human respiratory tract (Bogaert et al., 2004a; O’Brien et al., 2009; Welte et al., 2012; World Health Organization [WHO], 2019). Infants, toddlers and older adults are the age groups at highest risk of pneumococcal CAP and invasive pneumococcal disease (IPD) (Jansen et al., 2009), with young children considered to be the primary reservoir of pneumococci in the population (Bogaert et al., 2004b; Satzke et al., 2013). To reduce the burden of pneumococcal disease the World Health Organization (WHO) recommended in 2007 the implementation of pneumococcal conjugate vaccines (PCVs) (World Health Organization [WHO], 2007). Widespread introduction of PCVs into infant immunization programs has substantially reduced vaccine serotype disease (World Health Organization [WHO], 2019). Through herd immunity, PCV immunization of children has also reduced transmission of vaccine serotypes and the burden of pneumococcal disease in unvaccinated groups (Whitney et al., 2003). However, the overall impact of the PCVs implementation on pneumococcal disease has been limited by serotype replacement and aging populations (Lewnard and Hanage, 2019; van der Linden et al., 2019).

Pneumococcal carriage is now an accepted endpoint in vaccination studies (Dagan et al., 1997; van Gils et al., 2009; Flasche et al., 2011; Auranen et al., 2013), and surveillance of carriage is an important tool in monitoring the direct and indirect effects of vaccination. The laboratory protocol currently recommended by the WHO as the gold standard for pneumococcal carriage detection is the isolation of live pneumococci from culture of deep trans-nasal nasopharyngeal swab, complemented with an oropharyngeal swab for detection among adults (Satzke et al., 2013). However, this procedure lacks sensitivity when applied to highly polymicrobial samples in which pneumococcus is not a dominant bacterium (Heffron, 1939; Trzcinski et al., 2013; Krone et al., 2014; Wyllie et al., 2014, 2016a; Miellet et al., 2020; Wrobel-Pawelczyk et al., 2022) or to detect co-carriage of a secondary serotype (Huebner et al., 2000).

Pneumococcus was first described after having been isolated from saliva by both Sternberg and Pasteur separately (Pasteur, 1881; Sternberg, 1881). In early 20th century studies, saliva was also the preferred specimen for detection of pneumococcal carriers wherein high pneumococcal carriage prevalence rates across all ages were observed when saliva or oral washes were used by mouse inoculation (Heffron, 1939; Krone et al., 2014). However, with the advent of selective culture plates, nasopharyngeal swabs became the specimen of choice for pneumococcal detection and from that moment onward adult carriage rates abruptly declined in comparison with historical rates (Krone et al., 2014).

More recently, molecular diagnostic methods such as quantitative PCR (qPCR) have been proposed to improve the sensitivity of pneumococcal carriage surveillance across all ages (Carvalho et al., 2007; Trzcinski et al., 2013; Tavares et al., 2019). Studies from our group (Trzcinski et al., 2013; Wyllie et al., 2014, 2016a,b; Krone et al., 2015; Miellet et al., 2020), and others (Branche et al., 2018; Tavares et al., 2019; Almeida et al., 2020) have validated and applied pneumococcal carriage detection by qPCR in raw [minimally processed (MP)] and culture-enriched respiratory samples, demonstrating high sensitivity of *S. pneumoniae* detection (Miellet et al., 2022). Importantly, we (Wyllie et al., 2017) and others (Carvalho et al., 2012, 2013; Boelsen et al., 2020) have cautioned that assays used for molecular detection and serotyping of pneumococcus may exhibit reduced specificity in oral samples due to the presence of pneumococcal genes among commensal oral streptococci. Consequently, a robust experimental strategy is needed to maintain high specificity of pneumococcal detection in polymicrobial samples (Miellet et al., 2023). In a recent study we have described quantifying and comparing in an agreement analysis (Ranganathan et al., 2017) pneumococcal genes, *piaB* and *lytA*, and serotype/serogroup-specific genes in nasopharyngeal samples from children, and nasopharyngeal and oropharyngeal samples from adults (Miellet et al., 2022). This dual-target (or “Two-To-Tango”) approach ensures specific detection of pneumococcus by qPCR in polymicrobial samples and addresses specificity concerns of qPCR detection in oral samples. In children, the method demonstrated near-perfect agreement with conventional culture and was superior to culture when applied to samples from adults, which often exhibit low density carriage and positivity for pneumococcal carriage largely limited to oral samples (Miellet et al., 2022).

In the current study we evaluated saliva testing for carriage surveillance and vaccine studies and propose a protocol that enhances the specificity of molecular methods for the detection of live pneumococcus in oral fluids. Results from saliva were compared with results based on applying the same protocol in paired nasopharyngeal and oropharyngeal samples (Miellet et al., 2022). Inter-laboratory reproducibility of the protocol was tested independently in the second center. In conclusion, molecular testing of culture-enriched saliva samples improves the sensitivity of overall surveillance of pneumococcal carriage in children and adults, but limitations of qPCR-based approaches for pneumococcal serotypes carriage detection should be taken into account. The results highlight the importance of qPCR-based testing of oral samples.

## Materials and methods

### Study design and ethics statement

Pneumococcal carriage was investigated in cross-sectional prospective observational study conducted in 2015/2016 in the Netherlands (Vissers et al., 2018). The study was approved by the Medical Ethics Committee Noord Holland (NCT02522546 on clinicaltrials.gov). Written informed consent was obtained from the parent or guardian of every participating child, and adults produced written consent for their own participation. The study was conducted in accordance with the Declaration of Helsinki and Good Clinical Practice.

### Sample collection and laboratory processing

The collection and laboratory processing of nasopharyngeal and oropharyngeal swabs have been previously described (Vissers et al., 2018; Miellet et al., 2022). In brief, respiratory samples were collected between October 2015 and March 2016 in a study coordinated by the National Institute of Public Health and the Environment. Nasopharyngeal samples were collected from children aged 24 months (+1 month) and 44 to 49 months, all vaccinated with 10-valent pneumococcal vaccine (PHiD-CV, GlaxoSmithKline), and from parents of 24-month-olds (one parent per child). Oropharyngeal swabs were collected exclusively from unvaccinated adults (Watt et al., 2004; Vissers et al., 2018). Nasopharyngeal and oropharyngeal swabs were collected in accordance with standard procedures recommended by the WHO (Satzke et al., 2013). In addition, saliva samples were collected from all individuals as previously described (Krone et al., 2015). In short, oral fluids were collected with sponge lollipop (Oracol Saliva Collection System Malvern Medical Developments Limited, Worcester, UK), immediately transferred to tubes pre-filled with 100 μl sterile 50% glycerol solution in water, mixed, placed on dry ice and transported to the lab. With approximately 400 μl of saliva collected per sample the final glycerol concentration was around 10%. Saliva samples were delivered to the laboratory and stored at −70°C within 8 h.

### Detection of *S. pneumoniae*

Detection of *S. pneumoniae* and pneumococcal serotypes in nasopharyngeal and oropharyngeal samples, including qPCR-guided culturing, has been detailed previously (Miellet et al., 2022, 2023). Saliva samples stored frozen with 10% glycerol were thawed and diluted in an equal volume of PBS. Two-hundred microliters was used to inoculate SB7-GENT agar (Oxoid). After overnight incubation at 37°C and 5% CO_2_, all growth was harvested from a plate into 10% glycerol in BHI (Oxoid) as previously described for nasopharyngeal and oropharyngeal samples (Miellet et al., 2022) and stored at −70°C. We considered these samples culture-enriched for pneumococci. DNA was extracted from 200 μl of MP saliva samples diluted in PBS using DNeasy Blood and Tissue Kit (Qiagen) and eluted with 200 μl of buffer. We consider these templates to represent MP saliva. To extract DNA from culture-enriched saliva, 100 μl of a plate harvest was centrifuged for 2 min at 14,000 × *g*, the pellet was resuspended with 90 μl of the TE buffer [20 mM Tris–HCl (pH 8.0), 2 mM EDTA] and incubated for 15 min at 95°C. Next, 90 μl of lysis buffer [20 mM Tris–HCl (pH 8.0), 2 mM EDTA, 2.4% Triton X-100 and 40 mg/ml lysozyme] was added, and the samples were processed with DNeasy Blood & Tissue Kit and eluted with 200 μl of buffer (Miellet et al., 2020). Pneumococcal DNA was detected using a dual-target approach via single-plex qPCRs with primers and probes specific for regions within genes encoding for pneumococcal iron uptake ABC transporter lipoprotein PiaB (Trzcinski et al., 2013), and for major pneumococcal autolysin LytA (Carvalho et al., 2007) by using 5.5 μl eluate from MP or 1.0 μl for culture-enriched samples in a qPCR reaction volume of 12.5 μl.

### Molecular detection of pneumococcal serotypes

DNA extracts from culture-enriched samples were used to determine serotype composition of respiratory samples. We used 29 sets of primers and probes (Azzari et al., 2010, 2012; Pimenta et al., 2013; Velusamy et al., 2020) targeting 50 serotypes and including 24 vaccine serotypes covered by pneumococcal vaccines available in the Netherlands, namely ten-valent PHiD-CV, thirteen-valent PCV13 (Pfizer), and 23-valent polysaccharide vaccine PPV23 (Merck Sharp & Dohme). The panel also targeted a selection of non-vaccine serotypes, namely serotypes 6C, 6D, 7A, 9A, 9L, 10B, 11D, 12A, 12B, 15A, 15C, 15F, 18A, 18B, 18F, 16F, 21, 22A, 22F, 23A, 23B, 33A, 34, 35B, 35C, 37, and 38. With several qPCR assays it was not possible distinguish between serotypes of a serogroup, specifically 6A and 6B; 6C and 6D; 7A and 7F; 9A, 9L, 9N and 9V; 10A and 10B; 11A and 11D; 12A, 12B and 12F; 15A, 15B, 15C and 15F; 18A, 18B, 18C and 18F; 22A and 22F; 33A, 33F and 37; and 35B and 35C. Primers and probes used in these assays and their concentrations are listed in Supplementary Information. We employed a sample pooling strategy as described previously (Miellet et al., 2022). Namely samples with a C_*q*_ < 40 for *piaB* or *lytA* were pooled in groups of 5 and remaining samples were pooled in groups of 10. Negative samples were used to evaluate specificity of serotype-specific qPCRs. Pooled positive samples generating a signal for a serotype-specific qPCR assay were tested individually.

### Assessment of method’s inter-laboratory reproducibility

Two-hundred and twenty-nine culture-enriched saliva samples were randomly selected to evaluate the inter-laboratory reproducibility of molecular methods. For this, aliquots of samples from *n* = 133 children and *n* = 96 adults were shipped to the study site in England and tested as described above. Results for paired samples were compared between centers by calculating the percent agreement and Cohen’s kappa (κ). Quantitative results of both laboratories were also compared by calculating an intraclass correlation coefficient (ICC) and by comparing results in Bland–Altman plots. Carriage rates between both laboratories were compared using Cohen’s kappa.

### Definitions

For determination of C_*q*_ thresholds two different criteria were used for ROC curve analysis. The first criterion was based on the isolation of viable pneumococcus from primary diagnostic or qPCR-guided cultures and on quantification of *piaB* and *lytA* in saliva. Samples of C_*q*_ ≥ 40 for either *piaB* or *lytA* were regarded as negative when calculating maximum Youden index values. The second criterion was based solely on amplification slopes considered representing viable pneumococci in saliva. Here, we disregarded samples with no increase in both *piaB* and *lytA* C_*q*_s after culture-enrichment when compared with MP saliva. The first criterion can be applied to both MP and to culture-enriched samples. The second is applicable exclusively to culture-enriched samples yet requires MP samples to be tested.

For the evaluation of diagnostic test performance, we also used two different approaches. First, qPCR-based detection was compared with culture (primary diagnostic culture plus qPCR-guided culture) as reference standard for presence of carriage. We considered it to represent an imperfect reference (Naaktgeboren et al., 2013). The second was a composite study reference based on isolation of viable pneumococcus from a person (positivity by culture) or qPCR-based detection in saliva sample.

For evaluation of serotyping methods, qPCR-based serotyping was compared with culture (primary diagnostic culture only), and thereafter a composite reference standard with an any positive rule was also used to compare both culture (primary diagnostic culture) and qPCR-based serotyping of culture-enriched samples, including qPCR-based serotyping on nasopharyngeal samples for children and both, nasopharyngeal and oropharyngeal samples for adults. Comparisons between qPCR-based serotyping and culture were limited to qPCR-targeted serotypes. For serogroup-specific qPCR assays results were considered concordant when a serogroup detected in qPCR matched the serogroup of the serotype detected by culture.

### Statistical analysis

Analysis of carriage data was performed in GraphPad Prism software version 9.3.1 and R version 4.2.2. We performed ROC curve analysis with the “cutpointr” R package. Maximum Youden index values were estimated with bootstrapping (*n* = 5,000) on *piaB* or *lytA* qPCR data to determine C_*q*_ thresholds for qPCR detection (Nutz et al., 2011; Miellet et al., 2022). We used Bland–Altman plots (Bland and Altman, 1986; Ranganathan et al., 2017) with the “blandr” R package and two-way mixed effects ICCs (Koo and Li, 2016) with the “irr” R package to evaluate agreement between qPCR targets (Miellet et al., 2022).

Cohen’s kappa (κ) were calculated as described by McHugh (2012) and interpreted according to Landis and Koch (1977) with values of ≤0, 0.01–0.20, 0.21–0.40, 0.41–0.60, 0.61–0.80, and ≥0.81 interpreted as displaying poor, slight, fair, moderate, substantial, and near-perfect agreement, respectively (Miellet et al., 2022). The McNemar’s test was used to compare carriage rates unless otherwise stated. Diagnostic test performance was conducted as described previously (Miellet et al., 2022) and we compared diagnostic accuracy estimates between methods (or subgroups) using a test of interaction (Altman and Bland, 2003). A *p*-value of <0.05 was regarded as significant.

## Results

Samples collected from 653 children aged between 2 and 4 years and 318 adults were used to evaluate the diagnostic accuracy and added value of saliva testing for the detection of *S. pneumoniae* and pneumococcal serotypes carriage. For this, a paired comparison of saliva with nasopharyngeal samples from children, and with nasopharyngeal and oropharyngeal samples from adults was performed. Results for nasopharyngeal samples from children and adults and oropharyngeal samples from adults have previously been published (Miellet et al., 2022).

### Detection of pneumococcus in saliva

**FIGURE 1:**
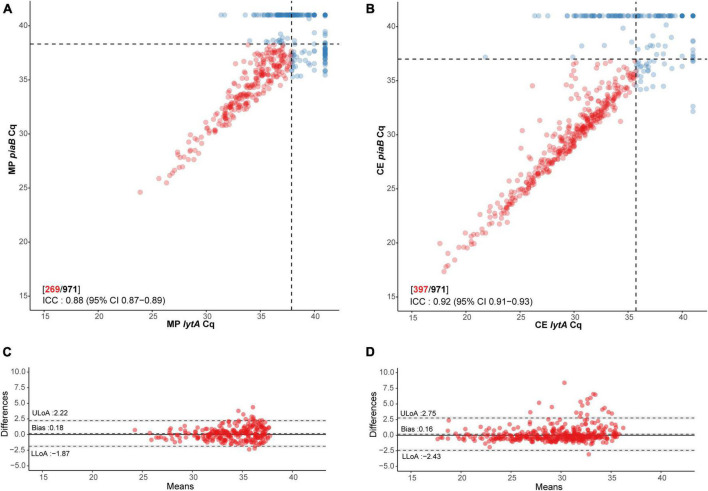
Scatter plot illustrating extent of agreement between *piaB* and *lytA* in **(A)** minimally processed and **(B)** culture-enriched saliva samples and Bland–Altman plots (**C,D**, respectively) displaying extent of agreement and bias among samples with C_*q*_s < 40 for *piaB* and *lytA*. Dots colored in red depict samples with C_*q*_s < 40 for *piaB* and *lytA*, remaining samples are colored in blue. Black dashed lines in scatter plots indicate data-driven thresholds. In Bland–Altman plots black dashed lines indicate the upper (ULoA) and lower limit of agreement (LLoA) and mean difference (bias). Shaded areas indicate 95% confidence interval. The solid black line indicates the line of equality (no bias).

The specificity of qPCR-based detection in saliva was enhanced by using a dual-target approach with *piaB* and *lytA* (Figure 1; Miellet et al., 2022). qPCR cycle threshold (C_*q*_) cut-off values were determined using ROC curve analysis with as criterion individuals that were previously determined positive by nasopharyngeal culture, or also positive by oropharyngeal culture for adults, and whose paired saliva samples yielded qPCR measurements <40 C_*q*_ (Table 1). To ascertain that a C_*q*_ cut-off reduced relic DNA presence and was likely to improve specificity of qPCR for detection of live pneumococci, the slopes of pneumococcal abundances in paired MP and culture-enriched samples were compared. ROC analysis was repeated with amplifying slopes as reference (Supplementary Figure 1 and Supplementary Table 1). Newly derived C_*q*_ cut-offs were similar to C_*q*_ cut-offs based on culture as reference (κ 0.96, 95% CI 0.94–0.98), the latter of which were used for further analysis.

**TABLE 1:** Optimal qPCR cycle threshold C_q_ for corresponding method for *Streptococcus pneumoniae* carriage detection in *n* = 971 saliva samples from children (*n* = 653) and adults (*n* = 318) samples.

Method	Criterion*	Optimal threshold *piaB* (95% CI)	Youden index *piaB*	Sensitivity *piaB*	Specificity *piaB*	Optimal threshold *lytA* (95% CI)	Youden index *lytA*	Sensitivity *lytA*	Specificity *lytA*
Minimally processed saliva	Culture and <40 C_*q*_s in sample	38.32 (37.17–38.60)	0.77	0.97	0.80	37.90 (37.09–38.70)	0.70	0.93	0.77
Culture-enriched saliva		37.01 (34.98–38.61)	0.68	0.97	0.72	35.73 (33.47–37.69)	0.61	0.94	0.67

Results from qPCR were validated in a receiver operating characteristic curve analysis based on isolation of viable pneumococcus from primary diagnostic culture or qPCR-guided culture and quantification of *piaB* and *lytA* in saliva. *A sample was considered positive when the following criterion has been fulfilled: (I) C_*q*_s for both *piaB* and *lytA* were <40 in the saliva sample tested with qPCR and (II) viable *S. pneumoniae* was recovered from either primary diagnostic or qPCR-guided culture of nasopharyngeal or (in case of adults) oropharyngeal samples collected from the person.

Subsequently, 336 samples from children (51.5% of 653) and 101 samples from adults (31.8% of 318) were identified as positive for pneumococcus by qPCR in either MP or culture-enriched saliva. In comparison, 368 (56.4% of 653) children and 22 (6.9% of 318) adults were positive by qPCR in either MP or culture-enriched nasopharyngeal samples. The proportions of samples positive for pneumococcus was significantly higher among culture-enriched saliva compared with MP saliva (397 or 40.9% vs. 269 or 27.7% of 971; *P* < 0.0001). Likewise, the proportions of pneumococcus positive samples were also significantly higher among children when compared with adults with MP saliva (226/653 or 34.6% vs. 43/318 or 13.5%; Fisher’s exact test *P* < 0.0001) and culture-enriched saliva (306/653 or 46.9% vs. 91/318 or 28.6%; *P* < 0.0001). Positive MP and culture-enriched saliva samples exhibited significant correlation between *piaB* and *lytA* C_*q*_s (Figure 1) and ICCs were indicative of excellent quantitative agreement of detection (0.93, 95% CI 0.91–0.94 and 0.95, 95% CI 0.93–0.96, respectively).

### Comparison between saliva and nasopharyngeal samples collected from children

**FIGURE 2:**
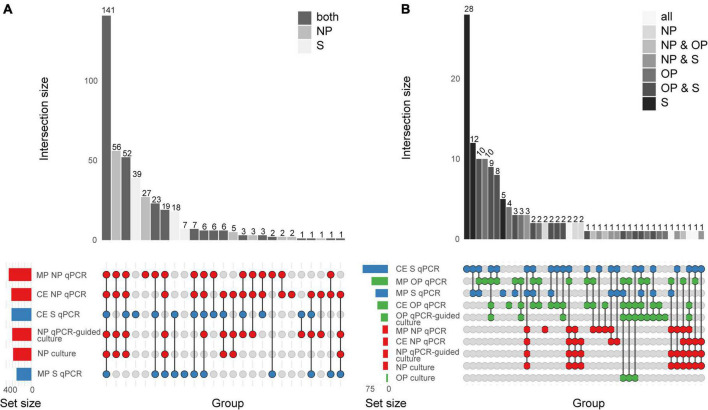
Quantitative PCR-based detection of *Streptococcus pneumoniae* among saliva and nasopharyngeal (NP) samples from **(A)** young children (*n* = 653) and **(B)** qPCR-based detection of *S. pneumoniae* among saliva (S), nasopharyngeal (NP), and oropharyngeal (OP) from adults (*n* = 318). The intersection bar diagrams display the total number of individuals positive per intersection. The matrix diagram displays intersection components (method or criterion). The set size bar diagram displays the total number of individuals positive per intersection component. Saliva, oropharyngeal, and nasopharyngeal samples are colored blue, green, and red, respectively, in the matrix diagram. “MP” stands for minimally processed, “CE” stands for culture-enrichment. “qPCR” stands for samples identified as positive for pneumococcus according with molecular method of qPCR and applying study criteria for positivity. “Culture” stands for isolation of viable *S. pneumoniae* from either primary diagnostic, or qPCR-guided culture.

The diagnostic accuracy of qPCR-based detection of pneumococcus in saliva samples from children was evaluated using primary diagnostic and qPCR-guided culturing as reference (Miellet et al., 2022). Both MP and culture-enriched saliva exhibited limited agreement with nasopharyngeal culture (Supplementary Table 2). In spite of similar pneumococcal detection rates with qPCR-based testing of culture-enriched saliva and detection by nasopharyngeal culture, there was limited overlap in positive samples (Figure 2A). Nineteen percent and thirty-eight percent of pneumococci detected were unique to nasopharyngeal cultures and culture-enriched saliva, respectively. Importantly, culture-enriched saliva samples displayed enhanced sensitivity when compared with MP saliva samples (*P* < 0.001).

Since nasopharyngeal detection (both by culture and qPCR) can be considered an imperfect study reference, the diagnostic accuracy of saliva testing was also evaluated using a composite study reference by which a result was considered to be true positive when positive by qPCR in culture-enriched saliva or positivity by nasopharyngeal culture. Testing culture-enriched saliva exhibited increased agreement with the composite study reference and enhanced sensitivity of detection when compared with nasopharyngeal cultures (*P* < 0.05, Table 2). Importantly, the ICC of positive culture-enriched saliva samples for which paired nasopharyngeal samples were negative for pneumococcus exhibited excellent quantitative agreement between *piaB* and *lytA* C_*q*_s (*n* = 87, ICC 0.93, 95% CI 0.89–0.95). Similar observations for performance of qPCR-based detection in culture-enriched saliva were made using C_*q*_ thresholds based on amplification slopes (Supplementary Table 3).

**TABLE 2:** The accuracy of *Streptococcus pneumoniae* detection in paired nasopharyngeal and saliva samples from *n* = 653 children in the Netherlands tested using molecular methods applied to DNA extracted from minimally processed and culture-enriched samples and applying *^ROCd^*C_q_ thresholds for sample positivity in qPCRs.

Method	Reference	Percent (*n*) of positive samples (95% CI)	PPV % (95% CI)	NPV % (95% CI)	Sensitivity % (95% CI)	Specificity % (95% CI)	Concordance % (95% CI)	κ (95% CI)
Primary nasopharyngeal culture	Composite reference	42.9 (280) (39.1–46.7)	100 (98.6–100)	70.2 (65.4–74.7)	71.6 (66.9–75.9)	100 (98.6–100)	83.0 (79.9–85.7)	0.67 (0.61–0.73)
Minimally processed saliva		34.6 (226) (31.1–38.3)	95.6 (92.0–97.6)	59.0 (54.3–63.6)	55.2 (50.3–60.1)	96.2 (93.1–97.9)	71.7 (68.1–75.0)	0.47 (0.40–0.53)
Culture-enriched saliva		46.9 (306) (43.1–50.7)	100 (98.8–100)	75.5 (70.7–79.7)	78.3 (73.9–82.1)	100 (98.6–100)	87.0 (84.2–89.3)	0.74 (0.69–0.79)

Measures of diagnostic accuracy were calculated using a composite study reference consisting of positivity in either qPCR-based *S. pneumoniae* detection on culture-enriched saliva sample or based on isolation of live pneumococcus either from the primary diagnostic or qPCR-guided nasopharyngeal culture. PPV, positive predictive value; NPV, negative predictive value; 95% CI, 95% confidence interval; κ, Cohen’s kappa where ≤0, 0.01–0.20, 0.21–0.40, 0.41–0.60, 0.61–0.80, and ≥0.81 are interpreted as no agreement, none to slight, fair, moderate, strong, and almost perfect agreement, respectively.

### Comparison between saliva and nasopharyngeal and oropharyngeal samples collected from adults

When comparing saliva samples to nasopharyngeal and oropharyngeal cultures (Figure 2B), limited agreement was observed among adults (Supplementary Table 4). Overall, testing culture-enriched saliva exhibited enhanced agreement with references when compared with MP saliva samples from adults by improving sensitivity of detection. Here, a composite study reference by which a result was considered true positive when positive by qPCR in culture-enriched saliva or positivity by nasopharyngeal or oropharyngeal culture, also indicated that saliva testing improved sensitivity of pneumococcal detection (*P* < 0.0001, Table 3). The ICC between *piaB* and *lytA* C_*q*_s for qPCR-positive culture-enriched saliva samples for which paired nasopharyngeal and oropharyngeal samples were negative for pneumococcus displayed good quantitative agreement (*n* = 60, ICC 0.79, 95% CI 0.68–0.87). Comparable performance of qPCR-based detection with culture-enriched saliva was made using C_*q*_ thresholds based on amplification slopes (Supplementary Table 5).

**TABLE 3:** The accuracy of *Streptococcus pneumoniae* detection of serotypes in paired nasopharyngeal, oropharyngeal, and saliva samples from *n* = 318 adults in the Netherlands tested using molecular methods applied to DNA extracted from minimally processed and culture-enriched samples and applying *^ROCd^*C_q_ thresholds for sample positivity in qPCRs.

Method	Reference	Percent (*n*) of positive samples (95% CI)	PPV % (95% CI)	NPV % (95% CI)	Sensitivity % (95% CI)	Specificity % (95% CI)	Concordance % (95% CI)	κ (95% CI)
Primary nasopharyngeal culture	Composite reference	4.7 (15) (2.9–7.6)	100 (79.6–100)	70.3 (64.9–75.2)	14.3 (8.9–22.2)	100 (98.2–100)	71.7 (66.5–76.4)	0.18 (0.04–0.33)
Primary oropharyngeal culture		0.9 (3) (0.3–2.7)	100 (43.9–100)	67.6 (62.3–72.5)	2.9 (1.0–8.1)	100 (98.2–100)	67.9 (62.6–72.8)	0.04 (−0.12 to 0.19)
Either primary nasopharyngeal or primary oropharyngeal culture		5.7 (18) (3.6–8.8)	100 (82.4–100)	71.0 (65.6–75.8)	17.1 (11.1–25.5)	100 (98.2–100)	72.6 (67.5–77.2)	0.22 (0.08–0.36)
Minimally processed saliva		13.5 (43) (10.2–17.7)	81.4 (67.4–90.3)	74.5 (69.1–79.3)	33.3 (25.0–42.8)	96.2 (92.8–98.1)	75.5 (70.5–79.9)	0.35 (0.22–0.47)
Culture-enriched saliva		28.6 (91) (23.9–33.8)	100 (95.9–100)	93.8 (89.9–96.3)	86.7 (78.9–91.9)	100 (98.2–100)	95.6 (92.7–97.4)	0.90 (0.84–0.95)

Measures of diagnostic accuracy were calculated using a composite study reference consisting of positivity in either qPCR-based *S. pneumoniae* detection on culture-enriched saliva sample or based on isolation of live pneumococcus either from the primary diagnostic or qPCR-guided nasopharyngeal or oropharyngeal culture. PPV, positive predictive value; NPV, negative predictive value; 95% CI, 95% confidence interval; κ, Cohen’s kappa where ≤0, 0.01–0.20, 0.21–0.40, 0.41–0.60, 0.61–0.80, and ≥0.81 are interpreted as no agreement, none to slight, fair, moderate, strong, and almost perfect agreement, respectively.

### Molecular detection of pneumococcal serotypes in culture-enriched samples of saliva

A pooling strategy was used to test culture-enriched saliva samples in 29 serotype- or serogroup-specific qPCR assays. Using pools of samples negative for *piaB* and *lytA*, we observed that assays targeting serotypes 4, 5, 17F, and 21 and serogroups 9, 12, 33, and 35 lack specificity (Supplementary Figure 2). However, for qPCRs targeting serotypes 21, 23A, and serogroup 33, Bland–Altman analysis indicated that there was sufficient agreement between signal for serotype and pneumococcus to consider results reliable and include in analysis (Supplementary Figure 3). For qPCRs targeting serotypes 4, 5, and 17F and serogroups 9, 12, and 35, there was no such agreement and all results generated in these six assays were excluded. With this, we analyzed results generated in twenty-three qPCRs targeting thirty-eight *S. pneumoniae* serotypes. Of those 23 assays, no sample of culture-enriched saliva tested positive with qPCR for serotype 2, 23F and serogroup 18, finding in line with results reported previously for paired nasopharyngeal and oropharyngeal samples (Miellet et al., 2022). Nevertheless, the presence of one or more serotype was detected in 90.2% (358 of 397) culture-enriched saliva samples classified as positive for pneumococcus. It represented 89.5% (274/306) and 92.3% (84/91) of such a sample from children and adults, respectively.

**FIGURE 3:**
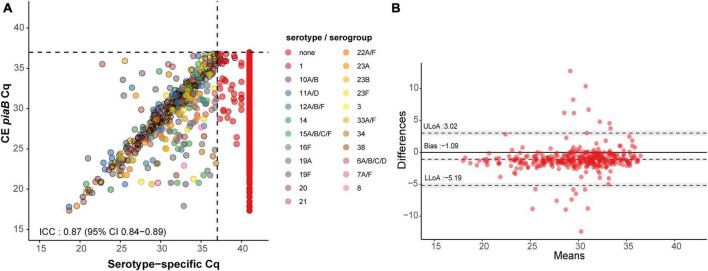
Scatter plot **(A)** illustrating extent of agreement between *piaB* and serotype/serogroup-specific detection among culture-enriched saliva samples (*n* = 353) and Bland–Altman plot **(B)** displaying extent of agreement and bias among samples with C_*q*_s < *^ROCd^*C_q_ for *piaB* and serotype/serogroup-specific qPCR assays. In panel **(A)**, saliva samples are colored by serotype/serogroup-specific signal detected. Samples classified as negative for any of the tested serotype/serogroup-specific qPCR assays are colored in red. Black dashed lines indicate data-driven *^ROCd^*C_q_ thresholds. In panel **(B)** (Bland–Altman plot) the degree of agreement between *piaB* and *lytA* C_q_ measurements among positive samples is shown. The mean difference in measurements is indicated by a dashed gray line and the standard deviations of the mean, upper limit of agreement (ULoA) and lower limit of agreement (LLoA) are also shown. Shaded areas indicate the 95% confidence interval. The solid black line indicates the line of equality (no bias) and dots above this line are for samples of which *lytA* C_*q*_s where lower than *piaB* C_*q*_s. ICC values <0.50, 0.50–0.75, 0.75–0.90, and ≥0.90 are indicative of poor, moderate, good, and quantitative agreement, respectively.

**FIGURE 4:**
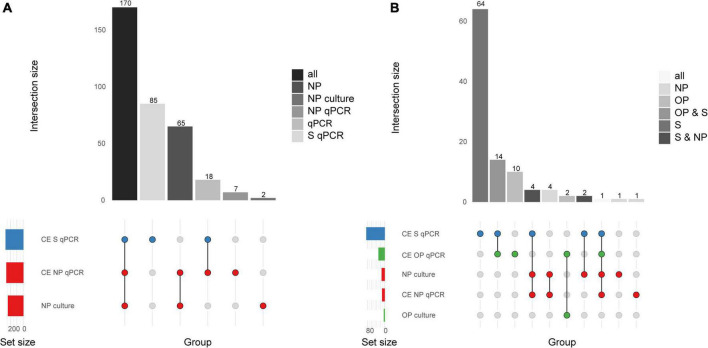
Quantitative PCR-based serotyping *Streptococcus pneumoniae* positive saliva (S) and nasopharyngeal (NP) samples from **(A)** young children (*n* = 653) and **(B)** qPCR-based serotyping of *S. pneumoniae* positive saliva, nasopharyngeal, oropharyngeal (OP) samples from adults (*n* = 318). The intersection bar diagrams display the total number of individuals positive per intersection. The matrix diagram displays intersection components (method or criterion). The set size bar diagram displays the total number of individuals positive per intersection component. Saliva, oropharyngeal, and nasopharyngeal samples are colored blue, green, and red, respectively, in the matrix diagram.

In total 463 serotype positives were observed among culture-enriched saliva samples. Serotypes that were ranked as dominant serotype within a sample displayed good agreement (ICC 0.87, 95% CI 0.84-0.89) with *piaB* C_*q*_s (Figure 3). Multiple serotype carriage was observed in 25.5% (78/306) and 13.2% (12/91) of positive culture-enriched samples from children and adults, respectively. The frequency of multiple serotype carriage was significantly higher among saliva samples when compared with nasopharyngeal samples (McNemar’s test, *P* < 0.0001, 22.7 vs. 9.3% or for saliva and nasopharyngeal samples, respectively). When evaluating the diagnostic accuracy of qPCR-based serotyping of culture-enriched saliva samples from children compared to nasopharyngeal cultures (Supplementary Table 6), testing saliva exhibited limited agreement with nasopharyngeal samples (Figure 4A). Using a composite study reference, testing of saliva samples exhibited increased sensitivity of detection when compared with nasopharyngeal cultures (*P* < 0.001) and increased agreement with the reference (Table 4). Among adults testing of saliva also exhibited limited agreement (Supplementary Table 7) to nasopharyngeal samples (Figure 4B). Saliva testing among adults was associated with significantly enhanced sensitivity of detected when compared to any other method (*P* < 0.0001, Figure 4B and Table 5).

**TABLE 4:** The accuracy of *Streptococcus pneumoniae* serotypes detection in paired nasopharyngeal and saliva samples from *n* = 653 children in the Netherlands tested using molecular methods applied to DNA extracted from minimally processed and culture-enriched samples and applying *^ROCd^*C_q_ thresholds for sample positivity in qPCRs.

Method	Reference	PPV % (95% CI)	NPV % (95% CI)	Sensitivity % (95% CI)	Specificity % (95% CI)	Concordance % (95% CI)	κ (95% CI)
Nasopharyngeal culture	Serotype composite reference	100 (98.4–100)	73.5 (69.0–77.5)	68.4 (63.3–73.1)	100 (98.8–100)	83.2 (80.1–85.8)	0.67 (0.61–0.73)
Culture-enriched NP qPCR		100 (98.5–100)	77.6 (73.2–81.5)	74.7 (69.9–79.0)	100 (98.8–100)	86.5 (83.7–88.9)	0.73 (0.68–0.79)
Culture-enriched saliva qPCR		100 (98.6–100)	80.5 (76.2–84.2)	78.7 (74.1–82.7)	100 (98.8–100)	88.7 (86.0–90.9)	0.78 (0.73–0.82)

Measures of diagnostic accuracy were calculated using a composite study reference consisting of number of samples in which the dominant serotype detected with either qPCR-based serotyping in culture-enriched nasopharyngeal or saliva sample or based on isolation of live pneumococcus from primary diagnostic nasopharyngeal culture. All serotypes detected by culture are considered dominant serotypes. PPV, positive predictive value; NPV, negative predictive value; 95% CI, 95% confidence interval; κ, Cohen’s kappa where ≤0, 0.01–0.20, 0.21–0.40, 0.41–0.60, 0.61–0.80, and ≥0.81 are interpreted as no agreement, none to slight, fair, moderate, strong, and almost perfect agreement, respectively.

**TABLE 5:** The accuracy of *Streptococcus pneumoniae* serotypes detection in paired nasopharyngeal and saliva samples from *n* = 318 adults in the Netherlands tested using molecular methods applied to DNA extracted from culture-enriched samples and applying *^ROCd^*C_q_ thresholds for sample positivity in qPCRs.

Method	Reference	PPV % (95% CI)	NPV % (95% CI)	Sensitivity % (95% CI)	Specificity % (95% CI)	Concordance % (95% CI)	κ (95% CI)
NP culture	Serotype composite reference	100 (75.8–100)	70.6 (65.3–75.4)	11.8 (6.9–19.4)	100 (98.3–100)	71.7 (66.5–76.4)	0.15 (0.01–0.30)
OP culture		100 (34.2–100)	68.4 (63.0–73.2)	2.0 (0.5–6.9)	100 (98.3–100)	68.6 (63.3–73.4)	0.03 (−0.13 to 0.18)
Culture-enriched NP qPCR		100 (72.2–100)	70.1 (64.8–75.0)	9.8 (5.4–17.1)	100 (98.3–100)	71.1 (65.9–75.8)	0.13 (−0.02 to 0.28)
Culture-enriched OP qPCR		100 (87.5–100)	74.2 (68.9–78.9)	26.5 (18.9–35.8)	100 (98.3–100)	76.4 (71.5–80.7)	0.33 (0.20–0.46)
Culture-enriched saliva qPCR		100 (95.6–100)	92.3 (88.2–95.1)	82.4 (73.8–88.5)	100 (98.3–100)	94.3 (91.2–96.4)	0.86 (0.80–0.92)

Measures of diagnostic accuracy were calculated using a composite study reference consisting of samples in which the dominant serotype detected with either qPCR-based serotyping in culture-enriched nasopharyngeal, oropharyngeal or saliva sample, or based on isolation of live pneumococcus from primary diagnostic nasopharyngeal or oropharyngeal culture. All serotypes detected by culture are considered dominant serotypes. PPV, positive predictive value; NPV, negative predictive value; 95% CI, 95% confidence interval; NP, nasopharyngeal; OP, oropharyngeal; κ, Cohen’s kappa where ≤0, 0.01–0.20, 0.21–0.40, 0.41–0.60, 0.61–0.80, and ≥0.81 are interpreted as no agreement, none to slight, fair, moderate, strong, and almost perfect agreement, respectively.

### Assessment of method’s inter-laboratory reproducibility

In order to evaluate the reproducibility of qPCR-based detection in culture-enriched saliva, 229 culture-enriched saliva samples were processed as part of an inter-laboratory comparison in both England and the Netherlands. We observed excellent quantitative agreement for *piaB* and *lytA* qPCRs between both laboratories, and near-perfect agreement in identifying culture-enriched saliva samples as positive for *S. pneumoniae* (κ 0.84, 95% CI 0.77–0.91). There was, however, reduced agreement between serotype/serogroup-specific assays (κ 0.40, 95% CI 0.28–0.52) that was driven by assays targeting serogroups 15A/B/C/F, 18A/B/C/F, and 33A/F/37 when performed in England. Exclusion of these assays resulted in substantial agreement between both laboratories (κ 0.68, 95% CI 0.58–0.77).

## Discussion

In the current study we sought to compare *S. pneumoniae* detection methods and characterize the diagnostic accuracy of qPCR-based saliva testing for carriage surveillance and vaccine studies in children and in adults. Saliva testing was compared with results based on applying the same protocol in paired nasopharyngeal and oropharyngeal samples, and inter-laboratory reproducibility of the protocol was evaluated independently in the second laboratory. Molecular testing of culture-enriched saliva samples improves the sensitivity of surveillance of pneumococcal carriage in children and adults, but there are also certain limitations of qPCR-based approaches for pneumococcal serotypes carriage detection to be considered. The protocol we propose enhances the specificity of molecular methods for the detection of live pneumococcus in highly polymicrobial samples.

Detection of pneumococcus by qPCR exhibited excellent quantitative agreement between *piaB* and *lytA* C_*q*_s in MP and culture-enriched saliva, demonstrating that reliable detection of pneumococcus in saliva is feasible. To further improve the specificity of qPCR-based detection of live pneumococci, we applied ROC-derived C_q_ cut-offs using nasopharyngeal and oropharyngeal cultures (for adults) as criterion as previously described (Miellet et al., 2022). The application of culture-enrichment, and ROC-derived C_q_ cut-offs reduced the frequency of qPCR positive samples which were attributable to quantification of relic DNA. This notion was confirmed by a comparison of qPCR quantification in paired MP and culture-enriched saliva samples. A comparison with a composite study reference indicated that testing of culture-enriched saliva samples enhances the sensitivity of *S. pneumoniae* detection when compared with conventional culture and nasopharyngeal sample testing. Notably, qPCR-based serotyping of saliva samples demonstrated superior sensitivity when compared with Quellung and resulted in significantly higher observed rates of multiple serotype carriage. However, we have also confirmed several constraints of the current qPCR-based approach for detection of certain serotypes due to the presence of the targeted sequences among non-pneumococcal bacterial species present in the saliva.

Adult pneumococcal carriers are seldom positive for *S. pneumoniae* in nasopharyngeal samples and are often exclusively positive for *S. pneumoniae* in oral samples. This discrepancy between nasopharyngeal and oral samples among adults was first noted Webster and Hughes (1931), and has also been observed in contemporary pneumococcal carriage studies (Trzcinski et al., 2013; Wyllie et al., 2016a; Miellet et al., 2022; Wrobel-Pawelczyk et al., 2022). Accordingly, testing of multiple sampling sites can improve the accuracy of carriage detection (Trzcinski et al., 2013; Krone et al., 2015; Wyllie et al., 2016a; Almeida et al., 2020, 2021; Miellet et al., 2022; Wrobel-Pawelczyk et al., 2022). However, contemporary carriage studies have repeatedly shown that conventional culture displays insufficient sensitivity when coupled with oral samples (Trzcinski et al., 2013; Krone et al., 2015; Wyllie et al., 2016a; Miellet et al., 2022; Wrobel-Pawelczyk et al., 2022), such as oropharyngeal swabs, as low pneumococcal abundance and commensal bacterial flora in oral samples can obscure pneumococcal presence with culture-based methods.

These limitations of conventional culture can be addressed with qPCR-based methods. We have previously evaluated qPCR-based nasopharyngeal sample testing and observed near-perfect agreement between qPCR and conventional culture (Miellet et al., 2022). We also noted that complementing primary culture with qPCR-guided culturing can greatly increase the number of adults from whom live pneumococci are recovered (Trzcinski et al., 2013; Wyllie et al., 2016a; Miellet et al., 2022). Unlike conventional culture, qPCR-based methods are highly suited to oral samples. In line with previous studies (Wyllie et al., 2014; Krone et al., 2015; Miellet et al., 2020), culture-enrichment of saliva significantly enhanced the sensitivity of detection. When qPCR detection was applied to culture-enriched instead of MP saliva samples the number of child and adult carriers identified increased by 41 and 111%, respectively. It underlines the importance of testing oral samples for sensitive surveillance of pneumococcal carriage among adults (Miellet et al., 2023).

Testing of saliva samples increased the number of carriers identified among children and adults, highlighting the benefits of testing multiple sampling sites. These observations confirm previous findings (Wyllie et al., 2014; Krone et al., 2015) and mirror pneumococcal prevalence rates from the early 20th century (Webster and Hughes, 1931; Heffron, 1939). When using a composite reference to accommodate the aforementioned observations, saliva testing exhibited high diagnostic accuracy when culture-enrichment was conducted prior to qPCR-based detection. When compared with the gold standard method, qPCR applied to culture-enriched saliva samples displayed significantly enhanced sensitivity of detection for both children and adults. Application of molecular methods to saliva samples allows us to capture an image of the *S. pneumoniae* carriage that mirrors the accuracy of nasopharyngeal sample testing in children and is superior in adults (Miellet et al., 2023). However, 19 and 37% of pneumococci detected by qPCR were unique to nasopharyngeal cultures and culture-enriched saliva samples, respectively. It implies that a negative nasopharyngeal sample does not necessarily preclude a positive saliva sample, and vice versa. As such, careful interpretation of carriage detection results is important (Miellet et al., 2023).

Prior to qPCR-based serotyping of individual samples, we evaluated the specificity of the method with pooled negative samples. The analysis indicated that qPCR assays for serotypes 4, 5, and 17F, and serogroups 9A/L/N/V, 12A/B, and 35B/C exhibited insufficient specificity in saliva samples. This was further confirmed by testing samples positive for *S. pneumoniae*, in which the signals for pneumococcus and for serotype were also discordant. In addition, Bland–Altman analysis indicated reduced specificity of assays targeting serotypes 21, 23A and serogroup 33A/F/37 in saliva. However, since there was concordance between serotype-specific quantification and *piaB* or *lytA* quantification, results for these assays were still considered reliable. After testing positive culture-enriched saliva samples with serotype-specific qPCRs, 37% of samples were positive for one or more serotypes in saliva. When compared with conventional culture but not qPCR-based nasopharyngeal sample testing, qPCR-based serotyping of saliva samples displayed superior sensitivity among both children and adults. Overall, we argue that application of molecular methods captures a reliable image of pneumococcal serotypes carriage (Miellet et al., 2023).

An important part of our study was an interlaboratory assessment of qPCR-based detection and serotyping of a subset of samples conducted in two laboratories. For detection of *S. pneumoniae* in culture-enriched saliva samples we observed near-perfect agreement between results generated in both centers. This is in line with results for nasopharyngeal and oropharyngeal samples from the same study (Miellet et al., 2022). However, with qPCR-based serotyping of culture-enriched saliva samples we observed limited agreement between results from England and the Netherlands. Reproducibility was affected by poor performance of certain assays and due to overrepresentation of weakly positive results which we attribute to minor differences between the two qPCR systems in the two laboratories. These results stress the importance of intra-laboratory controls, for instance by comparing concordance between *piaB* and serotype. Furthermore, these results also highlight the need for further multicenter validation of the protocol.

Several qPCR-based serotyping assays were observed to be unreliable in saliva samples, illustrating limitations of the method when applied to highly polymicrobial samples. Most importantly, qPCR assays targeting vaccine serotypes 4, 5, and 9V can be misinterpreted to represent circulation of VT *S. pneumoniae* in vaccinated children. However, we previously reported that assays targeting serotypes 4 and 5 were also unreliable in nasopharyngeal samples (Miellet et al., 2022), hence, this limitation of the qPCR is not unique to saliva alone. Similarly, increased richness of oral samples could impact the specificity of *lytA* and *piaB* qPCR assays. Whereas a number of studies report on *lytA* presence in non-pneumococcal streptococci (Kilian and Tettelin, 2019; Tavares et al., 2019; Gonzales-Siles et al., 2020), Tavares et al. is the only published study we are aware of to report on *piaB* detection in non-pneumococcal streptococcal strains. Importantly, neither of the two non-pneumococcal *Streptococcus* sp. strains reported by Tavares et al. (2019), to carry *piaB* (out of *n* = 433 tested), was positive for *lytA* gene. Consequently, although *lytA* and *piaB* can be simultaneously present in samples negative for *S. pneumoniae*, concordant quantification of both genes would be extremely rare. Of note, *piaB* is described by Gonzales-Siles et al. (2020) as *fepD* and by Kilian and Tettelin (2019) as SP_1033 and in both studies it is identified as one of only several genes unique to *S. pneumoniae*.

The study itself has certain limitations. First, the panel of serotype-specific and serogroup-specific qPCR did not cover all known one hundred and one pneumococcal serotypes. However, the serotypes not targeted by qPCR are rare in the Netherlands both in carriage and in IPD (Vissers et al., 2018). Secondly, with several qPCR assays targeting the serogroup rather than individual serotypes, we were not able to discern the serotype. This limitation might be of particular importance for groups that include both vaccine and non-vaccine serotypes.

Collectively, the study shows that testing of culture-enriched saliva samples improves the sensitivity of overall surveillance of pneumococcal carriage in children and adults. By ensuring the specificity of qPCR-based testing with a dual-target (or “Two-To-Tango”) approach and data-driven C_q_ thresholds, and evaluation of quantitative measurements with Bland–Altman analysis, pneumococcal detection in saliva mirrors the sensitivity of the gold standard in children. Moreover, qPCR-based serotyping in saliva samples from children outperforms the diagnostic accuracy of nasopharyngeal sample testing. Among adults, qPCR-based testing of oral samples provides high diagnostic accuracy unlike nasopharyngeal samples. These results demonstrate the importance of testing oral samples for sensitive surveillance of pneumococcal carriage. Finally, our results illustrate the limitations of the current gold standard method and emphasize the necessity of qPCR-based approaches for pneumococcal carriage detection.

## Data availability statement

The raw data supporting the conclusions of this article will be made available by the authors, without undue reservation.

## Ethics statement

The studies involving human participants were reviewed and approved by the Medical Ethics Committee Noord Holland. Written informed consent to participate in this study was provided by the participants’ legal guardian/next of kin.

## Author contributions

ES and KT had an idea and initiated the study. EM, NF, ES, and KT conceptualized the study. TB, NR, EM, NF, ES, and KT secured the financial support. KT led the project. AW-M, NR, and MH conducted the carriage study, collected the data, and provided the study materials. WM, JV, and KT developed and validated laboratory methods, wrote the laboratory protocol, and performed the formal analysis of study data. WM, JV, DL, RM, TN, RT, and SE analyzed the samples and collected the data. WM, RM, and KT contributed to the analytical tools. WM, JV, TN, RT, SE, and DL curated the data. WM, DL, RM, AW-M, TB, NF, and KT managed the study. WM and KT visualized the presentation of the results and drafted the manuscript. All authors amended, critically reviewed, and commented on the final manuscript.
