# Supplementary material for: A spitting image: molecular diagnostics applied to saliva enhance detection of Streptococcus pneumoniae and pneumococcal serotype carriage
Source: Front Microbiol. 2023 Apr 17;14:1156695. doi: 10.3389/fmicb.2023.1156695 (PMC10149683; doi:10.3389/fmicb.2023.1156695)
Supplement: Supplementary file 1 [file Data_Sheet_1.docx]

**Table S1.** Optimal qPCR cycle threshold C_q_ for *Streptococcus pneumoniae* carriage detection in saliva samples (n=971). Results from qPCR were validated in a receiver operating characteristic curve analysis with effect of culture-enrichment as criterion.

| **Method** | **Criterion*** | **Optimal threshold**  ***piaB***  (*95% CI*) | **Youden index**  ***piaB*** | **Sensitivity**  ***piaB*** | **Specificity**  ***piaB*** | **Optimal threshold**  ***lytA***  (*95% CI*) | **Youden**  **index**  ***lytA*** | **Sensitivity**  ***lytA*** | **Specificity**  ***lytA*** |
| --- | --- | --- | --- | --- | --- | --- | --- | --- | --- |
| culture-enriched saliva | amplifying slope | 38.03  *(36.79 – 39.49)* | 0.92 | 0.98 | 0.94 | 36.85  *(35.79 – 37.52)* | 0.83 | 0.97 | 0.85 |

NP – nasopharyngeal sample; OP – oropharyngeal sample; * – a sample was considered positive for the criterion when culture-enriched samples displayed an enrichment of *piaB* and *lytA* signal (‘amplifying slope’) when compared with paired minimally processed sample.

**Table S2.** The accuracy of ***Streptococcus pneumoniae* detection** in paired nasopharyngeal and saliva **samples from** **n=653 children** in the Netherlands tested using molecular methods applied to DNA extracted from minimally processed and culture-enriched samples and applying ^ROCd^C_q_ thresholds for sample positivity in qPCRs. Measures of diagnostic accuracy were calculated by comparing the number of detected samples positive per method with the number of individuals positive for *S. pneumoniae* based on isolation of live pneumococcus either from the primary diagnostic or qPCR-guided nasopharyngeal culture as imperfect reference. The accuracy of *S. pneumoniae* detection by qPCR with culture as reference was described in a previous publication for nasopharyngeal and oropharyngeal samples from this cohort [32].

| **Method** | **Reference** | **Percent (n) of positive samples**  ***(95%CI)*** | **PPV**  **%**  **(*95%CI*)** | **NPV**  **%**  **(*95%CI*)** | **Sensitivity %**  **(*95%CI*)** | **Specificity  %**  **(*95%CI*)** | **Concordance %**  **(*95%CI*)** | ***κ***  **(*95%CI*)** |
| --- | --- | --- | --- | --- | --- | --- | --- | --- |
| minimally processed saliva | culture | 34.6 (226)  *(31.1 – 38.3)* | 74.3  *(68.3 – 79.6)* | 70.3  *(65.8 – 74.4)* | 56.9  *(51.2 – 62.5)* | 83.8  *(79.6 – 87.3)* | 71.7  *(68.1 – 75.0)* | 0.42  *(0.34 – 0.49)* |
| culture-enriched saliva | culture | 46.9 (306)  *(43.1 – 50.7)* | 68.6  *(63.2 – 73.6)* | 75.5  *(70.7 – 79.7)* | 71.2  *(65.8 – 76.1)* | 73.2  *(68.4 – 77.5)* | 72.3  *(68.7 – 75.6)* | 0.44  *(0.37 – 0.51)* |

PPV – positive predictive value; NPV – negative predictive value; 95%CI – 95% confidence interval; *κ* – Cohen’s kappa where ≤0, 0.01-0.20, 0.21-0.40, 0.41-0.60, 0.61-0.80, ≥0.81 are interpreted as no agreement, none to slight, fair, moderate, strong, and almost perfect agreement, respectively.

**Table S3.** The accuracy of ***Streptococcus pneumoniae* detection** in paired nasopharyngeal and saliva **samples from** **n=653 children** in the Netherlands tested using molecular methods applied to DNA extracted from minimally processed and culture-enriched samples and applying ^ROCd^C_q_ thresholds for sample positivity in qPCRs with amplification slopes as criterion for ROC curve analysis (see table S1).

| **Method** | **Reference** | **Percent (n) of positive samples**  ***(95%CI)*** | **PPV**  **%**  **(*95%CI*)** | **NPV**  **%**  **(*95%CI*)** | **Sensitivity %**  **(*95%CI*)** | **Specificity  %**  **(*95%CI*)** | **Concordance %**  **(*95%CI*)** | ***κ***  **(*95%CI*)** |
| --- | --- | --- | --- | --- | --- | --- | --- | --- |
| primary nasopharyngeal culture | composite reference | 42.9 (280)  *(39.1 – 46.7)* | 100  *(98.6 – 100)* | 68.6  *(63.8 – 73.1)* | 70.5  *(65.9 – 74.8)* | 100  *(98.5 – 100)* | 82.1  *(79.0 – 84.8)* | 0.65  *(0.60 – 0.71)* |
| minimally processed saliva |  | 34.6 (226)  *(31.1 – 38.3)* | 96.5  *(93.2 – 98.2)* | 58.1  *(53.3 – 62.7)* | 54.9  *(50.0 – 59.7)* | 96.9  *(94.0 – 98.4)* | 71.4  *(67.8 – 74.7)* | 0.46  *(0.40 – 0.53)* |
| culture-enriched saliva |  | 48.9 (319)  *(45.0 – 52.7)* | 100  *(98.8 – 100)* | 76.6  *(71.8 – 80.9)* | 80.4  *(76.2 – 84.0)* | 100  *(98.5 – 100)* | 88.1  *(85.3 – 90.3)* | 0.76  *(0.71 – 0.81)* |

PPV – positive predictive value; NPV – negative predictive value; 95%CI – 95% confidence interval; *κ* – Cohen’s kappa where ≤0, 0.01-0.20, 0.21-0.40, 0.41-0.60, 0.61-0.80, ≥0.81 are interpreted as no agreement, none to slight, fair, moderate, strong, and almost perfect agreement, respectively.

**Table S4.** The accuracy of ***Streptococcus pneumoniae* detection** in paired nasopharyngeal, oropharyngeal and saliva **samples from** **n=318 adults** in the Netherlands tested using molecular methods applied to DNA extracted from minimally processed and culture-enriched samples and applying ^ROCd^C_q_ thresholds for sample positivity in qPCRs. Measures of diagnostic accuracy were calculated by comparing the number of detected samples positive per method with the number of individuals positive for *S. pneumoniae* based on isolation of live pneumococcus either from the primary diagnostic or qPCR-guided nasopharyngeal or oropharyngeal culture as imperfect reference. The accuracy of *S. pneumoniae* detection by qPCR with culture as reference was described in a previous publication for nasopharyngeal and oropharyngeal samples from this cohort [32].

| **Method** | **Reference** | **Percent (n) of positive samples**  ***(95%CI)*** | **PPV**  **%**  **(*95%CI*)** | **NPV**  **%**  **(*95%CI*)** | **Sensitivity %**  **(*95%CI*)** | **Specificity  %**  **(*95%CI*)** | **Concordance %**  **(*95%CI*)** | ***κ***  **(*95%CI*)** |
| --- | --- | --- | --- | --- | --- | --- | --- | --- |
| minimally processed saliva | culture | 13.5 (43)  *(10.2 – 17.7)* | 16.3  *(8.1 – 30.1)* | 89.1  *(84.9 – 92.3)* | 18.9  *(9.5 – 34.2)* | 87.2  *(82.8 – 90.6)* | 79.2  *(74.5 – 83.3)* | 0.06  *(-0.15 – 0.26)* |
| culture-enriched saliva |  | 28.6 (91)  *(23.9 – 33.8)* | 25.3  *(17.5 – 35.1)* | 93.8  *(89.9 – 96.3)* | 62.2  *(46.1 – 75.9)* | 75.8  *(70.5 – 80.4)* | 74.2  *(69.1 – 78.7)* | 0.23  *(0.09 – 0.38)* |

PPV – positive predictive value; NPV – negative predictive value; 95%CI – 95% confidence interval; *κ* – Cohen’s kappa where ≤0, 0.01-0.20, 0.21-0.40, 0.41-0.60, 0.61-0.80, ≥0.81 are interpreted as no agreement, none to slight, fair, moderate, strong, and almost perfect agreement, respectively.

**Table S5.** The accuracy of ***Streptococcus pneumoniae* detection** in paired nasopharyngeal, oropharyngeal and saliva **samples from** **n=318 adults** in the Netherlands tested using molecular methods applied to DNA extracted from minimally processed and culture-enriched samples and applying ^ROCd^C_q_ thresholds for sample positivity in qPCRs with amplification slopes as criterion for ROC curve analysis (see **Table S1**).

| **Method** | **Reference** | **Percent (n) of positive**  **samples**  ***(95%CI)*** | **PPV**  **%**  **(*95%CI*)** | **NPV**  **%**  **(*95%CI*)** | **Sensitivity %**  **(*95%CI*)** | **Specificity  %**  **(*95%CI*)** | **Concordance %**  **(*95%CI*)** | ***κ***  **(*95%CI*)** |
| --- | --- | --- | --- | --- | --- | --- | --- | --- |
| primary nasopharyngeal  culture | composite reference | 4.7 (15)  *(2.9 – 7.6)* | 100  *(79.6 – 100)* | 69.0  *(63.6 – 73.9)* | 13.8  *(8.5 – 21.5)* | 100  *(98.2 – 100)* | 70.4  *(65.2 – 75.2)* | 0.17  *(0.03 – 0.31)* |
| primary oropharyngeal  culture |  | 0.9 (3)  *(0.3 – 2.7)* | 100  *(43.9 – 100)* | 66.3  *(61.0 – 71.3)* | 2.8  *(0.9 – 7.8)* | 100  *(98.2 – 100)* | 66.7  *(61.3 – 71.6)* | 0.04  *(-0.11 – 0.19)* |
| either primary nasopharyngeal  or primary oropharyngeal  culture |  | 5.7 (18)  *(3.6 – 8.8)* | 100  *(82.4 – 100)* | 69.7  *(64.2 – 74.6)* | 16.5  *(10.7 – 24.6)* | 100  *(98.2 – 100)* | 71.4  *(66.2 – 76.1)* | 0.21  *(0.07 – 0.34)* |
| minimally processed saliva |  | 13.5 (43)  *(10.2 – 17.7)* | 81.4  *(67.4 – 90.3)* | 73.1  *(67.6 – 78.0)* | 32.1  *(24.1 – 41.4)* | 96.2  *(92.6 – 98.0)* | 74.2  *(69.1 – 78.7)* | 0.33  *(0.21 – 0.46)* |
| culture-enriched saliva |  | 30.5 (97)  *(25.7 – 35.8)* | 100  *(96.2 – 100)* | 94.6  *(90.8 – 96.9)* | 89.0  *(81.7 – 93.6)* | 100  *(98.2 – 100)* | 96.2  *(93.5 – 97.8)* | 0.91  *(0.87 – 0.96)* |

PPV – positive predictive value; NPV – negative predictive value; 95%CI – 95% confidence interval; *κ* – Cohen’s kappa where ≤0, 0.01-0.20, 0.21-0.40, 0.41-0.60, 0.61-0.80, ≥0.81 are interpreted as no agreement, none to slight, fair, moderate, strong, and almost perfect agreement, respectively.

**Table S6.** The accuracy of ***Streptococcus pneumoniae* serotypes detection** in paired nasopharyngeal and saliva **samples from** **n=653** **children** in the Netherlands tested using molecular methods applied to DNA extracted from culture-enriched samples and applying ^ROCd^C_q_ thresholds (see **Table 1**) for sample positivity in qPCRs. Measures of diagnostic accuracy were calculated by comparing the numbers of serotype carriage events detected with molecular methods with nasopharyngeal samples from which serotypes were cultured***** that were targeted by serotype-specific qPCR assays as imperfect reference.

| **Method** | **Reference±** | **PPV**  **%**  **(*95%CI*)** | **NPV**  **%**  **(*95%CI*)** | **Sensitivity %**  **(*95%CI*)** | **Specificity  %**  **(*95%CI*)** | **Concordance %**  **(*95%CI*)** | ***κ***  **(*95%CI*)** |
| --- | --- | --- | --- | --- | --- | --- | --- |
| culture-enriched NP qPCR | culture | 100  *(98.4 – 100)* | 98.6  *(96.9 – 99.3)* | 97.5  *(94.6 – 98.8)* | 100  *(99.1 – 100)* | 99.1  *(98.0 – 99.6)* | 0.98  *(0.96 – 1)* |
| culture-enriched saliva qPCR |  | 100  *(97.6 – 100)* | 83.8  *(80.3 – 86.8)* | 66.4  *(60.2 – 72.1)* | 100  *(99.1 – 100)* | 87.7  *(85.0 – 90.0)* | 0.72  *(0.66 – 0.77)* |

PPV – positive predictive value; NPV – negative predictive value; 95%CI – 95% confidence interval; *κ* – Cohen’s kappa where ≤0, 0.01-0.20, 0.21-0.40, 0.41-0.60, 0.61-0.80, ≥0.81 are interpreted as no agreement, none to slight, fair, moderate, strong, and almost perfect agreement, respectively; ***** – all serotypes detected by culture are considered dominant serotypes; ± – the reference was limited to primary diagnostic culture as all of primary diagnostic *S. pneumoniae* nasopharyngeal cultures have been subjected to Quellung-based serotyping.

**Table S7.** The accuracy of ***Streptococcus pneumoniae* serotypes** **detection** in paired nasopharyngeal and saliva **samples from** **n=318 adults** in the Netherlands tested using molecular methods applied to DNA extracted from culture-enriched samples and applying ^ROCd^C_q_ thresholds for a sample positivity in qPCRs. Measures of diagnostic accuracy were calculated by comparing the numbers of serotype carriage events detected with molecular methods with nasopharyngeal and oropharyngeal samples from which serotypes were cultured***** that were targeted by serotype-specific qPCR assays as imperfect reference.

| **Method** | **Reference±** | **PPV**  **%**  **(*95%CI*)** | **NPV**  **%**  **(*95%CI*)** | **Sensitivity %**  **(*95%CI*)** | **Specificity  %**  **(*95%CI*)** | **Concordance %**  **(*95%CI*)** | ***κ***  **(*95%CI*)** |
| --- | --- | --- | --- | --- | --- | --- | --- |
| NP culture* | culture | 100  *(75.8 – 100)* | 99.3  *(97.6 – 99.8)* | 85.7  *(60.1 – 96.0)* | 100  *(98.8 – 100)* | 99.4  *(97.7 – 99.8)* | 0.92  *(0.81 – 1)* |
| OP culture* |  | 100  *(34.2 – 100)* | 96.2  *(93.5 – 97.8)* | 14.3  *(4.0 – 39.9)* | 100  *(98.8 – 100)* | 96.2  *(93.5 – 97.8)* | 0.24  *(-0.18 – 0.66)* |
| culture-enriched NP qPCR |  | 100  *(70.1 – 100)* | 98.4  *(96.3 – 99.3)* | 64.3  *(38.8 – 83.7)* | 100  *(98.8 – 100)* | 98.4  *(96.4 – 99.3)* | 0.77  *(0.58 – 0.97)* |
| culture-enriched OP qPCR |  | 100  *(43.9 – 100)* | 96.5  *(93.9 – 98.0)* | 21.4  *(7.6 – 47.6)* | 100  *(98.8 – 100)* | 96.5  *(93.9 – 98.1)* | 0.34  *(-0.04 – 0.72)* |
| culture-enriched saliva qPCR |  | 100  *(61.0 – 100)* | 97.4  *(95.0 – 98.7)* | 42.9  *(21.4 – 67.4)* | 100  *(98.8 – 100)* | 97.5  *(95.1 – 98.7)* | 0.59  *(0.31 – 0.87)* |

PPV – positive predictive value; NPV – negative predictive value; 95%CI – 95% confidence interval; NP – nasopharyngeal; OP – oropharyngeal; *κ* – Cohen’s kappa where ≤0, 0.01-0.20, 0.21-0.40, 0.41-0.60, 0.61-0.80, ≥0.81 are interpreted as no agreement, none to slight, fair, moderate, strong, and almost perfect agreement, respectively; ***** – all serotypes detected by culture are considered dominant serotypes; ± – the reference was limited to primary diagnostic culture as all of primary diagnostic *S. pneumoniae* cultures have been subjected to Quellung-based serotyping.
