# Supplementary material for: A spitting image: molecular diagnostics applied to saliva enhance detection of Streptococcus pneumoniae and pneumococcal serotype carriage
Source: Front Microbiol. 2023 Apr 17;14:1156695. doi: 10.3389/fmicb.2023.1156695 (PMC10149683; doi:10.3389/fmicb.2023.1156695)
Supplement: Supplementary file 2 [file Data_Sheet_2.docx]

**
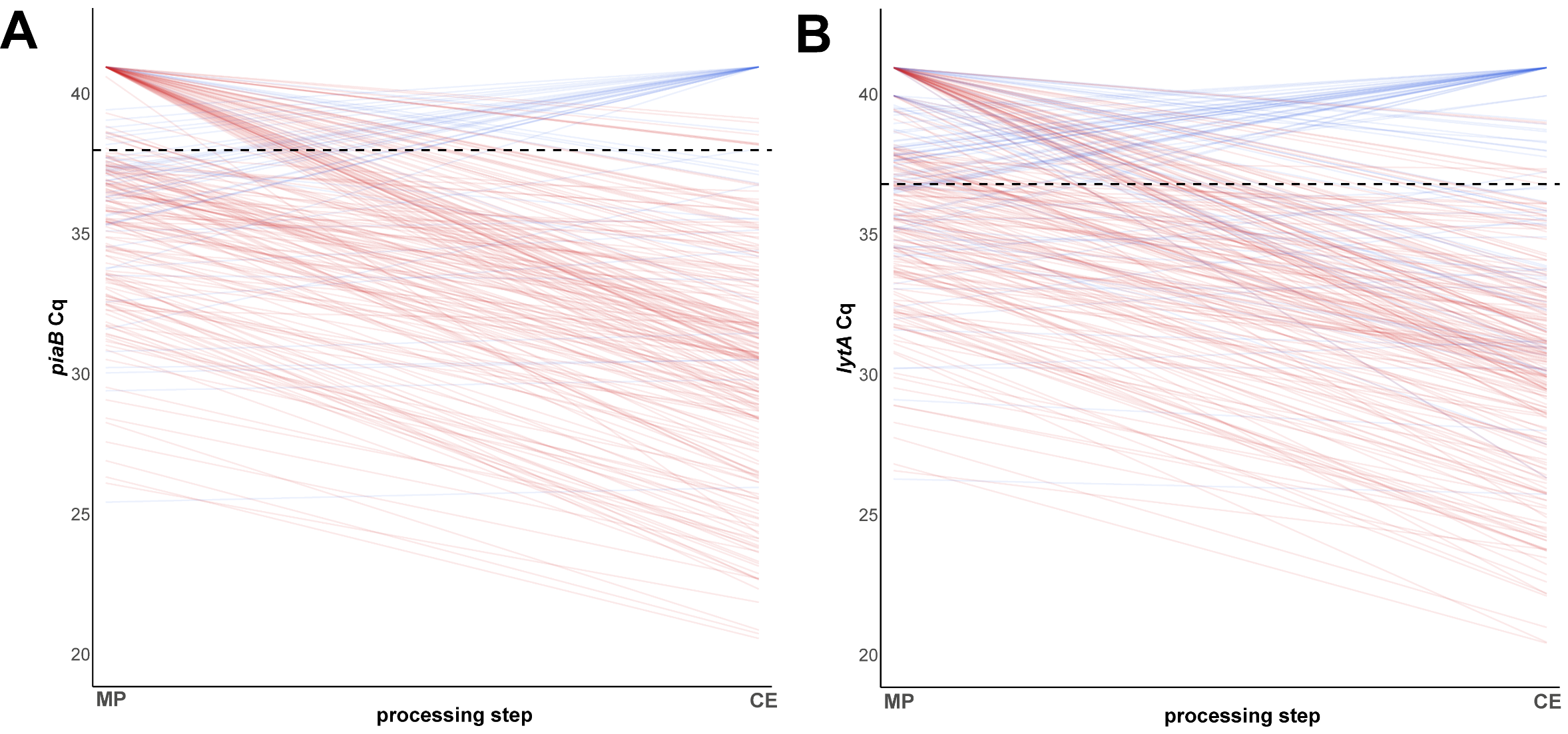
**

**Figure S1 : Plots illustrating amplification of *Streptococcus pneumoniae* presence after enrichment in saliva samples with *piaB* (A) and *lytA* (B) qPCRs.** Lines coloured in red indicate amplification of *piaB* or *lytA* signal and lines coloured in blue lines indicate lack of amplification. Black dashed lines indicate data-driven C_q_ thresholds with amplifying slopes of *piaB* or *lytA* C_q_ as reference (see table S1). The plots visualize potential presence of relic DNA, indicated by blue lines, in minimally processed (MP) and culture-enriched (CE) saliva samples.

**Figure S2.** **Detection of *Streptococcus pneumoniae* serotypes with molecular methods in culture-enriched samples of saliva.** The panels depict scatter plots of pneumococcus-specific (*piaB*) and serotype-specific (assays targeting individual serotypes 1, 2, 3, 4, 5, 8, 14, 16F, 17F, 19A, 19F, 20, 21, 23A, 23B, 23F, 34 and 38) or group-specific (assays targeting serotypes 6A/6B/6C/6D, 7A/7F, 9A/9L/9N/9V, 10A/10B, 11A/11D, 12A/12B/12F, 15A/15B/15C/15F, 18A/18B/18C/18F, 22A/22F, 33A/33F/37, and 35B/35C) quantification in twenty-nine different serotype/serogroup specific qPCRs. Each symbol (dot) represents an individual sample. Exception are dots of C_q_=41.00 for *piaB* representing samples negative for both, *lytA* and *piaB*, where symbol represents a pool of ten samples. Blue dots represent saliva samples from n=653 children. Green dots represent saliva samples from n=318 adults. Dark-colored dots represent saliva from individuals from which *S. pneumonia* strain of corresponding serotype was cultured from any sample tested (nasopharyngeal or saliva sample in case of children and nasopharyngeal, oropharyngeal or saliva sample in case of adults). Light-colored dots represent samples from individuals with no viable *S. pneumoniae* of the corresponding serotype recovered via culture. Red dashed lines mark the ^ROCd^C_q_ thresholds of 37.01 (**Table 1**) for sample positivity for *S. pneumoniae* (vertical lines) and for serotype/serogroup (horizontal lines). Samples with a C_q_ >40 in a particular serotype-specific or serogroup-specific qPCR are not depicted. Dotted diagonal lines represent a hypothetical line of perfect agreement between signals for *S. pneumoniae* and for a serotype/serogroup. Assays targeting serotypes **1**, **2***, **3**, **8**, **14**, **16F**, **19A**, **19F**, **20**, **23B**, **23F**, **34**, **38**, and serogroups **6**, **7**, **10**, **11**, **15**, **18***, **22** were classified as reliable based on agreement with signal detected for *S. pneumoniae*, negligible number of samples with a signal for serotype stronger than for S. *pneumonia**e,* and lack of any signal for serotype/serogroup in samples of Cq >40 for *piaB* and *lytA*. Despite signal for serotype being stronger than for *S. pneumoniae*, or being detected in samples negative for pneumococcus, assays targeting serotypes **21**, **23A**, and serogroup **33** were considered reliable based on sufficient agreement between C_q_ in samples positive for both, pneumococcus and serotype/serogroup (**Figure S3)**. Assays targeting serotypes **4**, **5**, **17F**, and serogroups **9**, **12** and **35** were classified as unreliable based on lack of sensitivity (qPCR assay targeting serotype **17F**) or specificity (all six qPCRs) as manifested by lack of correlation between signals for serotype and pneumococcus.

* - no single sample of culture-enriched saliva generated any signal in qPCR assays targeting serotype **2** and serogroup **18**, hence there is no panels depicting results for these two assays.

**
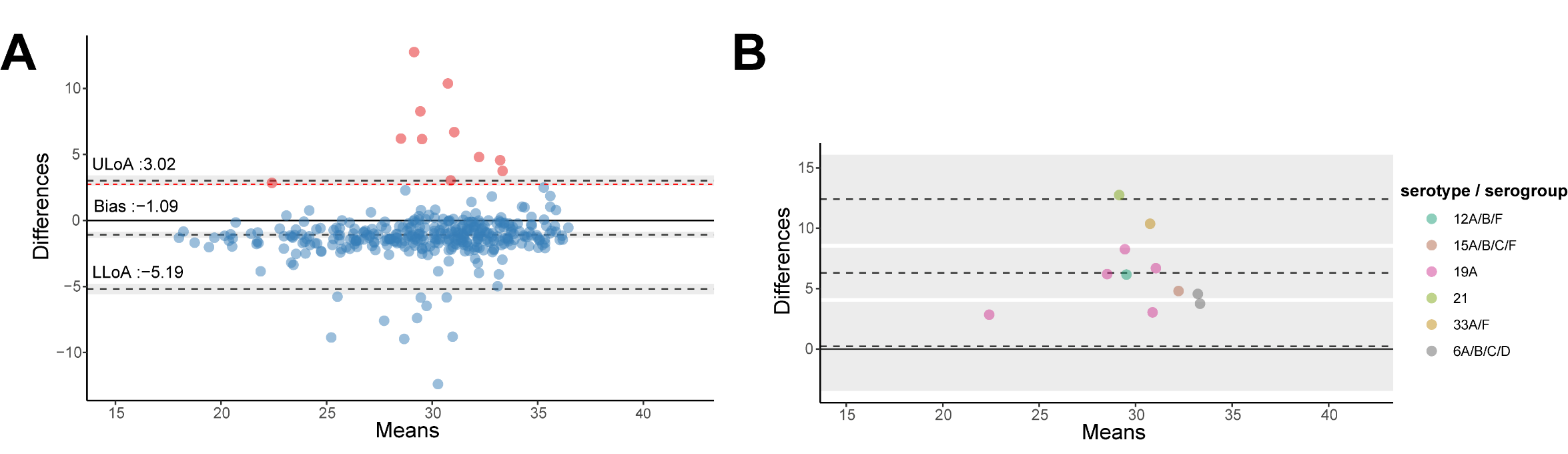
**

**Figure S3: Bland-Altman plot illustrating extent of agreement and bias between *piaB* and serotype/serogroup-specific qPCR assays to evaluate serotype/serogroup-specific assay quantitative agreement.** Samples coloured red in panel A display serotype/serogroup-specific measurements that exceed the *a priori* acceptable limit based on *piaB* and *lytA* agreement (limit of agreement). Only serotype/serogroups-specific qPCR assays measurements analyzed that were ranked as dominant in a sample. The red dashed line in panel is the *a priori* acceptable limit used to classify serotype-specific C_q_ results as specific, the limit is calculated from the upper limit of agreement between *piaB* and *lytA* C_q_s. In panel B only the outlier serotype C_q_ measurements are shown and the dots are coloured by serotype/serogroup-specific qPCR assay. Bland-Altman analysis can be used to identify outlier measurements and non-reliable assays. The degree of agreement between targets is shown. The mean difference in measurements is indicated by a dashed grey line and the standard deviations of the mean, upper limit of agreement (ULoA) and lower limit of agreement (LLoA) are also shown. Shaded areas indicate the 95% confidence interval. The solid black line indicates the line of equality (no bias) and dots above this line are for samples of which *lytA* C_q_s where lower than *piaB* C_q_s. ICC values <0.50, 0.50-0.75, 0.75-0.90 and >0.90 are indicative of poor, moderate, good, and quantitative agreement, respectively.
